# Supplementary material for: OpenMM 7: Rapid development of high performance algorithms for molecular dynamics
Source: PLoS Comput Biol. 2017 Jul 26;13(7):e1005659. doi: 10.1371/journal.pcbi.1005659 (PMC5549999; doi:10.1371/journal.pcbi.1005659)
Supplement: S1 Source Code — (ZIP) [file pcbi.1005659.s002.zip › OpenMM-7.0.1-Source/docs/api-python/index.html]

OpenMM Python API — OpenMM 7.0.1.dev-5e86c4f documentation


# OpenMM Python API¶

The Python API provides information about the classes and methods available in OpenMM for Python developers.

OpenMM consists of two parts. First, there is a set of libraries for performing many types of computations needed for molecular simulations: force evaluation, numerical integration, energy minimization, etc.

Second, there is an application layer, a set of Python libraries providing a high level interface for running simulations. This layer is targeted at computational biologists or other people who want to run simulations, and who may or may not be programmers.

See the user guide for more details.

- Application Layer
  - Loaders and Setup
  - Representation and Manipulation
  - Simulation
  - Reporting Output
  - Extras
- Library Layer
  - Core Objects
  - Forces
  - Integrators
  - Extras

High performance molecular simulation on GPUs


### Navigation

- Application Layer
- Library Layer

©2015, Stanford University and the Authors.
|
Powered by Sphinx 1.3.1
& Alabaster 0.7.7
|
Page source
